# Ions in the Deep Subsurface of Earth, Mars, and Icy Moons: Their Effects in Combination with Temperature and Pressure on tRNA–Ligand Binding

**DOI:** 10.3390/ijms221910861

**Published:** 2021-10-08

**Authors:** Nisrine Jahmidi-Azizi, Stewart Gault, Charles S. Cockell, Rosario Oliva, Roland Winter

**Affiliations:** 1 Physical Chemistry I-Biophysical Chemistry, Department of Chemistry and Chemical Biology, TU Dortmund University, 44227 Dortmund, Germany; nisrine.jahmidi@tu-dortmund.de; 2 UK Centre for Astrobiology, SUPA School of Physics and Astronomy, University of Edinburgh, James Clerk Maxwell Building, Edinburgh EH9 3FD, UK; s.a.gault@sms.ed.ac.uk (S.G.); c.s.cockell@ed.ac.uk (C.S.C.)

**Keywords:** Martian salts, high pressure, nucleic acid-ligand binding, tRNA, ThT, perchlorate

## Abstract

The interactions of ligands with nucleic acids are central to numerous reactions in the biological cell. How such reactions are affected by harsh environmental conditions such as low temperatures, high pressures, and high concentrations of destructive ions is still largely unknown. To elucidate the ions’ role in shaping habitability in extraterrestrial environments and the deep subsurface of Earth with respect to fundamental biochemical processes, we investigated the effect of selected salts (MgCl_2_, MgSO_4_, and Mg(ClO_4_)_2_) and high hydrostatic pressure (relevant for the subsurface of that planet) on the complex formation between tRNA and the ligand ThT. The results show that Mg^2+^ salts reduce the binding tendency of ThT to tRNA. This effect is largely due to the interaction of ThT with the salt anions, which leads to a strong decrease in the activity of the ligand. However, at mM concentrations, binding is still favored. The ions alter the thermodynamics of binding, rendering complex formation that is more entropy driven. Remarkably, the pressure favors ligand binding regardless of the type of salt. Although the binding constant is reduced, the harsh conditions in the subsurface of Earth, Mars, and icy moons do not necessarily preclude nucleic acid–ligand interactions of the type studied here.

## 1. Introduction

Revealing the mechanistic details of the formation, stability, and reactivity of biomolecular systems such as membranes, nucleic acids, and proteins under extreme environmental conditions, including high hydrostatic pressures and high concentrations of salts, is of fundamental biological, astrobiological, and biotechnological relevance [1,2,3,4]. In general, biopolymers can adopt several structural states, each with its own specific volume, and pressure acts to reshuffle the relative distribution of these states, favoring conformations with smaller volumes [5,6,7,8,9,10,11]. Moreover, by direct or water-mediated interactions, salt cations and anions can affect the conformation and hydration properties of biomolecules and hence their function [12,13].

While the effects of pressure on membranes and proteins have been extensively studied in recent years, the effects of temperature, pressure, and high salt concentrations on the conformation and stability of nucleic acids and their reactivity, such as ligand binding, are less explored, apart from a few works [5,6,14,15]. However, the canonical DNA duplex formed from Watson–Crick base pairs generally exhibits a negative partial molar volume, suggesting that the application of pressure causes the duplex to be more stable [11,16,17,18]. In contrast to the stability of DNA duplexes, non-canonical DNA structures, such as G-quadruplexes or i-motifs, have been found to be more sensitive to pressure [19,20,21,22]. Much less is known about how pressure affects the stability and reactivity of RNA structures. The RNA duplex containing AU base pairs is slightly destabilized by pressure, and pressure has been found to destabilize tetraloop–receptor docking [23,24]. Pressure-induced structural changes of tRNA^Phe^ have been shown to be quite small. Only about 15% unpaired bases have been observed upon pressurization up to 10 kbar (1 GPa) [25].

In recent years, increasing interest has been directed at how extreme conditions on other planetary bodies might influence the potential for those environments to support life. Even in the absence of life, we might ask whether the physical and chemical conditions to be found there would theoretically limit known biochemistry, in the process giving us fundamental general insights into the effects of chemical and physical extremes on biochemical limits.

The subsurface of Mars is thought to be more habitable [26] than the surface of the planet on account of the lack of liquid water and relatively high ionizing flux at the surface, yet Mars is known to have localized concentrations of salts, which include chlorides [27], sulfates [28], and perchlorates [29]. These observations motivate us to ask how high concentrations of these salts in combination with high pressure would affect basic biochemical processes, such as molecular recognition and binding. These insights, as well as informing us about the habitability of Mars, have implications for the habitability of the salty, high-pressure interior of the icy moons of Jupiter (e.g., Europa) and Saturn (e.g., Enceladus) and some asteroids such as Ceres, where sulfates and chlorides exist [30,31]. Furthermore, in the deep subsurface of Earth, high ion concentrations, such as sulfate and chloride ions [3], can occur in combination with high pressure, yet we know very little about how these conditions determine the required biochemical adaptations in life to reproduce in these extremes.

Herein, we studied the effect of temperature, ion concentration of mono and divalent salts, and high hydrostatic pressure on the ligand-binding properties of transfer RNA (tRNA) from *E. coli*. tRNAs are short noncoding RNAs, and typical cytoplasmic tRNAs are approximately 70–90 ribonucleotides long. The secondary structure of almost all tRNA molecules adopts an L-shaped cloverleaf-like structure, with the anticodon region at one end and the CCA acceptor sequence for the aminoacyl–tRNA synthetase at the other [32]. The tertiary structure of tRNA is maintained by extensive stacking interactions and base pairing within and between its helical stems, and many of the tertiary base-pairing interactions are non-Watson–Crick associations. Their structure is largely conserved in all domains of life, the eukarya, bacteria, and archaea, and function as adaptor molecules that act as links between mRNAs and the amino acid sequences of proteins. In most cases, protein binding to tRNA is mediated by three conserved residues, one Arg/Lys that forms a salt bridge to the phosphodiester backbone, and two aromatic residues that create stacking interactions with the nucleobases [33].

We used Thioflavin T (ThT; 4-(3,6-dimethyl-1,3-benzothiazol-3-ium-2-yl)-*N,N*-dimethylaniline chloride) as the ligand, which is a well-known fluorescence probe used for detecting amyloid fibrils. The substantial increase in the fluorescence emission upon binding is due to restricting the rotation, enforcing the planarization of the dye molecule. ThT has also been used as a specific fluorescent sensor for the human telomeric G-quadruplex, which is a four-stranded nucleic acid structure that is formed by the stacking of Hoogsteen base-paired coplanar guanines [34]. Recently, it was also shown that ThT can be used as an RNA-binding probe for monitoring RNA metabolism, both in vitro and in vivo [35]. In addition, the search for small molecule ligands for RNAs has identified mostly highly basic (and thus positively charged under physiological conditions) and planar molecules that can intercalate between and attach to RNA bases [36]. Some small positively charged ligands, with antibacterial properties (e.g., tobramycin), can bind tRNAs, inducing significant conformational changes as well [37]. Other neutral aromatic ligands have also been shown to bind to tRNA [38]. Finally, some drugs with antioxidant activity (e.g., safranal) are aromatic molecules capable of protecting tRNAs from harmful reactions [39]. Hence, we think that the ThT molecule harboring a positive charge and aromatic ring structure is an appropriate prototype ligand reminiscent of many tRNA-relevant binding motifs.

## 2. Results

**Figure 1:**
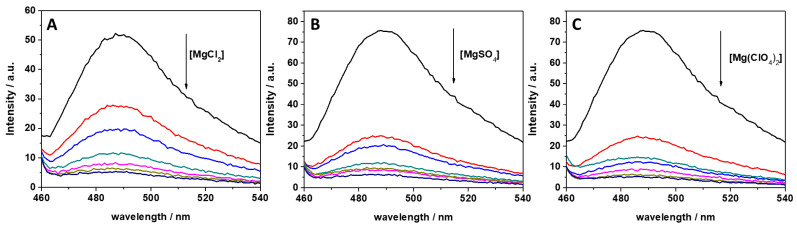
Fluorescence emission spectra of a 0.5 μM ThT solution, mixed with 100 μM of tRNA from *E. coli* in the presence of (**A**) MgCl_2_, (**B**) MgSO_4_, and (**C**) Mg(ClO_4_)_2_. The arrows indicate the direction of increasing concentration of the magnesium salt. Color code: black (0 mM Mg salt), red (0.5 mM Mg salt), blue (1 mM Mg salt), dark cyan (2 mM Mg salt), magenta (5 mM Mg salt), dark yellow (10 mM Mg salt), and navy (20 mM Mg salt). All the spectra were acquired in 20 mM Tris-HCl buffer, 40 mM KCl, pH 6.9, at the temperature of 25 °C and pressure of 1 bar. The intensity is reported in arbitrary units (a.u.).

In previous studies, it was reported that ThT has the ability to bind to several nucleic acid molecules, such as G-quadruplexes, DNAs, and RNAs [34,35,40]. ThT was also found to bind specifically to tRNA [34]. It was found that ThT is able to bind to tRNA with a binding constant of 5.5 × 10^4^ M^−1^ in Tris-HCl buffer in the presence of 40 mM K^+^ (pH 7.0), assuming a 1:1 binding stoichiometry. To reveal the impact of MgCl_2_, MgSO_4_, and Mg(ClO_4_)_2_ on the binding characteristics of ThT to tRNA, we performed a series of fluorescence experiments with the aim of quantitatively describing complex formation through the determination of the binding constant, *K*_b_. First, the impact of different concentrations of these salts on ThT binding was explored by recording fluorescence emission spectra of 0.5 μM ThT with 100 μM tRNA in the presence of the three salts in a concentration range between 0 and 20 mM. The fluorescence emission spectra are reported in Figure 1.

**Figure 2:**
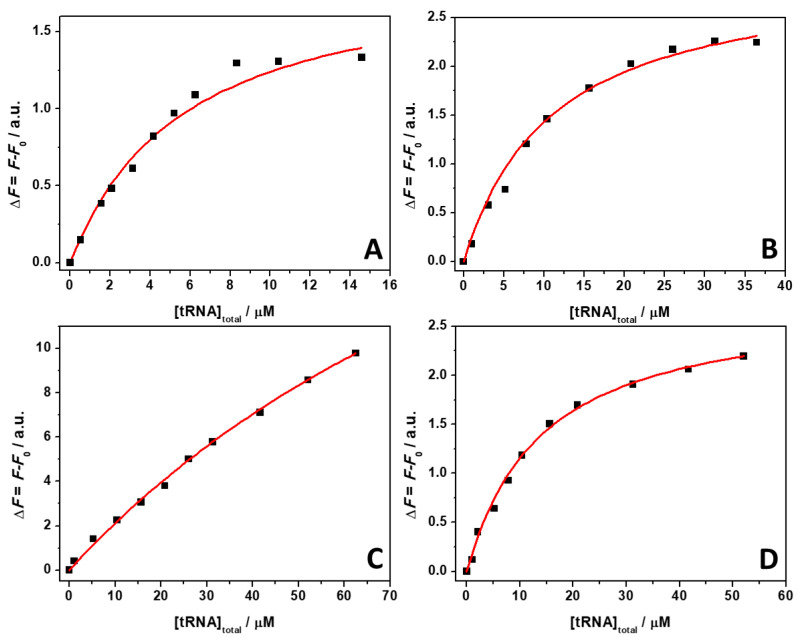
Binding isotherms for complex formation between tRNA and ThT obtained at the temperature of 25 °C and pressure of 1 bar in the absence (**A**) and in the presence of 1 mM of MgCl_2_ (**B**), MgSO_4_ (**C**), and Mg(ClO_4_)_2_ (**D**). The solid lines represent the best fit to the experimental data according to a 1:1 binding model. The binding isotherms were obtained by plotting Δ*F* = *F* − *F*_0_ vs. total concentration of tRNA. Here, *F* and *F*_0_ are the ThT fluorescence intensities in the presence and in the absence of tRNA, respectively. All the experiments were performed in 20 mM Tris-HCl buffer, 40 mM KCl, pH 6.9.

ThT emission is characterized by a very low quantum yield when it is free in solution [40]. Upon binding to nucleic acids, a strong increase of the fluorescence quantum yield is observed, which depends on the targeted structure. Thus, ThT is able to fluoresce essentially only in the complexed form; hence, the presence of fluorescence signal is indicative of binding. The spectra reported in Figure 1 clearly show that the fluorescence intensity of the bound ThT decreases with increasing salt concentration. Visual inspection of the data already suggests a rather weak binding of the ligand to the tRNA in the presence of Mg salts. However, the drop in the intensity depends on the type of salt present in solution. For example, at a 0.5 mM Mg salt concentration, the drop of fluorescence intensity with respect to the spectra in the absence of salts, is 47%, 66%, and 68% in the presence of MgCl_2_, MgSO_4_, and Mg(ClO_4_)_2_, respectively. Since the binding of ThT to tRNA is strongly affected by Mg salts already at low concentrations, the binding constants for the complex formation were determined in the presence of 1 mM magnesium salts. In these experiments, a solution with a fixed concentration of ThT (0.5 µM) was titrated with a solution of tRNA. The extent of complex formation was followed by recording the increase of the fluorescence intensity of ThT upon the addition of tRNA. The value of the binding constants can be determined by fitting the experimental data with a 1:1 binding model equation, as described previously [41]. Figure 2 depicts the binding isotherms obtained at ambient conditions (*T* = 25 °C and *p* = 1 bar), in the absence (Figure 2, panel A) and in the presence of 1 mM of MgCl_2_ (Figure 2, panel B), MgSO_4_ (Figure 2, panel C), and Mg(ClO_4_)_2_ (Figure 2, panel D). For comparison, the binding isotherm obtained in the presence of 150 mM NaCl is reported in Figure S1 (see Supplementary Materials). The numerical values of all binding constants determined are collected in Table 1.

The complex formation between ThT and tRNA at neat buffer conditions is characterized by a binding constant of 0.16 × 10^6^ M^−1^. Surprisingly, the addition of 150 mM NaCl has only a minor effect on the binding process. A small decrease of *K*_b_ was observed with respect to the pure buffer condition, only. Instead, when in the presence of the Mg salts, a significant decrease of the *K*_b_ values was observed. A decrease of *K*_b_ to 0.078 × 10^6^ M^−1^ was observed in the presence of MgCl_2_. In the presence of Mg(ClO_4_)_2_, *K*_b_ was found to be slightly lower (0.053 × 10^6^ M^−1^). Conversely, a drastic decrease of *K*_b_ was observed in the presence of the MgSO_4_, reaching a value of *K*_b_ = 0.009 × 10^6^ M^−1^, which can be due to the binding of sulfate to ThT, thereby hampering the interaction with tRNA (see the Discussion section). Collectively, these data highlight the ability of Mg salts to strongly modulate the complex formation between ThT and tRNA, which is a phenomenon that has been previously observed also for protein–ligand interactions [42].

**Figure 3:**
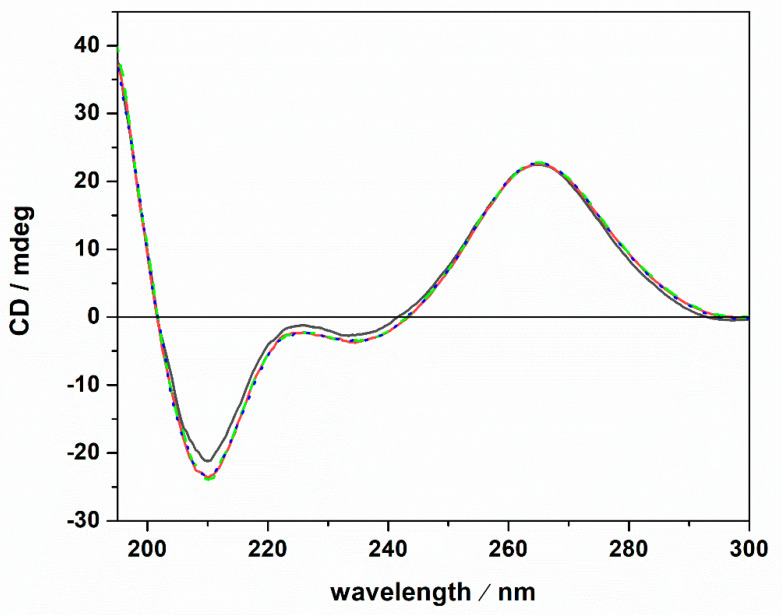
Circular dichroism spectra of a 20 μM tRNA solution in the absence (black spectrum) and in the presence of 1 mM MgCl_2_ (red spectrum), MgSO_4_ (dotted blue spectrum) and Mg(ClO_4_)_2_ (green dashed spectrum). All the experiments were carried out at the temperature of 25 °C in 20 mM Tris-HCl buffer, 40 mM KCl, pH 6.9, using a 0.1 cm path length quartz cuvette.

A possible cause of the observed *K*_b_ changes could be a conformational change of the tRNA imposed by the presence of the Mg salts. To test this possibility, circular dichroism (CD) spectra of tRNA were recorded. Figure 3 depicts CD spectra of tRNA in Tris-HCl buffer, 40 mM KCl, pH 6.9, and in the presence of Mg salts.

**Figure 4:**
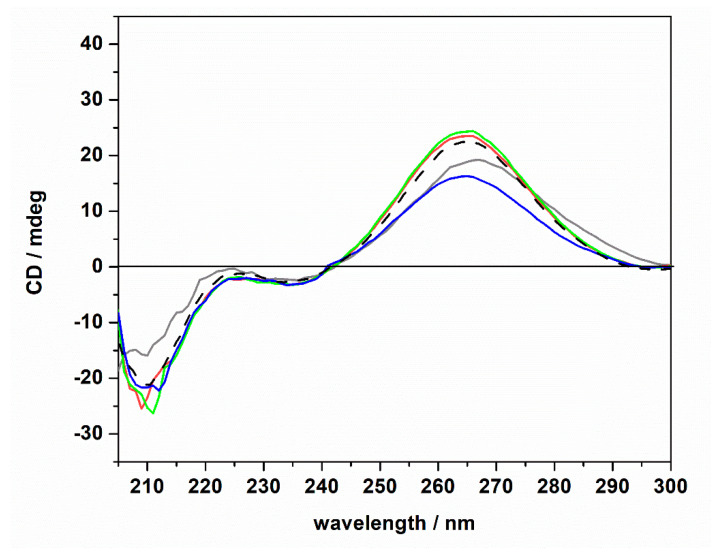
Circular dichroism spectra of a 20 μM tRNA solution in the presence of 1 mM of ThT dissolved in 20 mM Tris-HCl buffer, 40 mM KCl, pH 6.9, in the absence (gray spectrum) and in the presence of 1 mM of MgCl_2_ (red spectrum), MgSO_4_ (blue spectrum), and Mg(ClO_4_)_2_ (green spectrum). For comparison, the CD spectrum of tRNA in neat buffer in the absence of ThT is also reported (dashed black spectrum).

The CD spectrum of tRNA in neat buffer conditions (no Mg salts) is characterized by a negative band around 210 nm. Two further bands are present around 223 and 235 nm, and a strong positive band is centered around 265 nm. However, the intensity of the 223 nm band is quite low. These spectral features are indicative of a folded tRNA in the A conformation [38,43,44]. In fact, the relative intensities of the bands around 210 and 235 nm can be used to discriminate between A and B duplex forms [45]. In particular, for the A form, the intensity of the band at 210 nm is much higher with respect to the intensity of the band at 235 nm. For the B form, the opposite is observed [46]. Thus, tRNA adopts a folded A conformation in neat buffer conditions where 40 mM K^+^ cations are present. Generally, mono and divalent cations are needed to stabilize the folded tertiary structure of tRNAs [25,47,48]. A direct interaction of K^+^ ions with binding sites on N7 and O6 atoms on nucleobases has been reported [49]. Several Mg^2+^ ions can bind with high affinity to tRNA [47], interacting with phosphate groups of the nucleic acid backbone [49]. Of note, the tRNA employed in this study was extracted from *E. coli*. Thus, it is expected that some Mg^2+^ cations are already bound to the tRNA, contributing to the stability of the folded structure as inferred from the CD spectrum in pure buffer. Upon the addition of 1 mM magnesium chloride, sulfate, and perchlorate, no significant conformational changes were detected, indicating that the structural integrity of the tRNA’s A form is preserved, even in the presence of the three Mars-relevant salts used. In the next step, we explored the impact of ThT binding on the conformational behavior of the tRNA in neat buffer and in the presence of the Mg salts. The corresponding CD spectra are reported in Figure 4.

The CD spectra of tRNA (20 μM) in the presence of ThT (1 mM) were found to be qualitatively similar to those obtained in the absence of the ligand. Only small changes in the intensity of the positive band around 265 nm were detected (please note the shift of this band in the absence of magnesium, which is possibly due to a small conformational change of the tRNA structure). The general spectral features, i.e., the presence of the positive band at 265 nm, the two bands at around 223 and 235 nm, and the negative band at around 210 nm, suggest that the tRNA is still folded in the A form. Thus, we may conclude that even if some small (and local) conformational changes may take place upon ThT binding, the native folded structure of the tRNA is preserved upon ThT binding. Consequentially, the marked differences observed in the *K*_b_ data cannot be ascribed to any major conformational change of the tRNA molecule induced by the salts and/or upon ThT binding; i.e., other reasons need to be invoked to explain these findings (see Discussion section).

**Figure 5:**
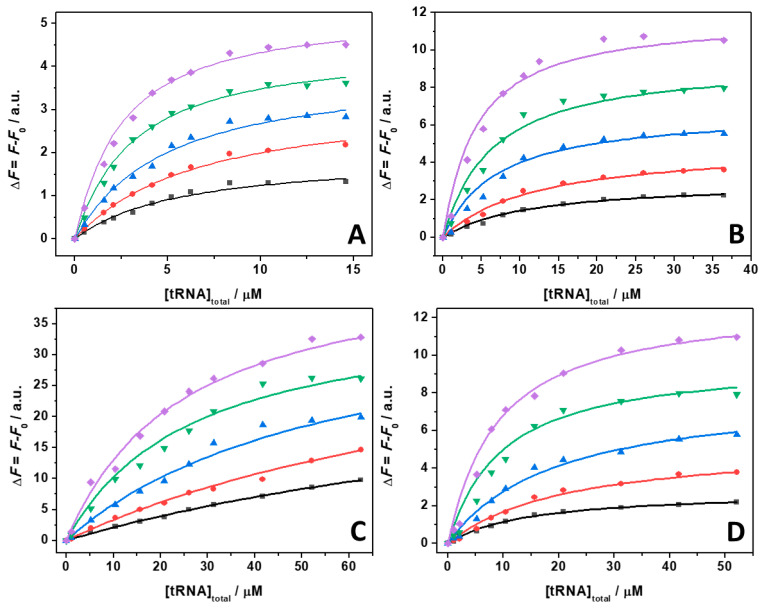
Binding isotherms for complex formation between tRNA and ThT obtained at the temperature of 25 °C in the absence (**A**) and in the presence of 1 mM of MgCl_2_ (**B**), MgSO_4_ (**C**), and Mg(ClO_4_)_2_ (**D**), at the following pressure values: 1 bar (black squares), 500 bar (red circles), 1000 bar (blue triangles), 1500 bar (green reversed triangles), and 2000 bar (magenta diamonds). The concentration of ThT was 0.5 µM. The solid lines represent the best fit to the experimental data according to a 1:1 binding model. The binding isotherms were obtained by plotting Δ*F* = *F* − *F*_0_ vs. total concentration of tRNA. Here, *F* and *F*_0_ are the ThT fluorescence intensities in the presence and in the absence of tRNA, respectively. All the experiments were performed in 20 mM Tris-HCl buffer, pH 6.9.

To shed more light on the intimate nature of the binding process, we also employed pressure dependent fluorescence spectroscopy, which provides information about the volumetric changes accompanying the binding event [50]. To this end, the values of the binding constants were determined in the pressure range between 1 and 2000 bar in the absence and in the presence of the Mg salts at 1 mM concentration. For comparison, the experiment was also performed in the presence of 150 mM NaCl (Figure S2). Figure 5 shows the binding isotherms for *T* = 25 °C. The corresponding *K*_b_ values determined by the fitting procedure are collected in Table 2.

The data reported in Table 2 reveal that in all cases, the application of pressure leads to an increase of the binding constant. In other words, the application of pressure favors the formation of the ThT–tRNA complex. A close inspection of Table 2 shows that the pressure-induced increase of the binding constants depends on the salt type used. At neat buffer conditions as well as in the presence of NaCl and MgCl_2_, a 3.1, 3.6, and 3.2 times increase of *K*_b_ was observed upon a pressure increase of 2 kbar. Instead, in the presence of the sulfate salt, the increase of *K*_b_ is 4.3 times. Conversely, in the presence of perchlorate, the increase of *K*_b_ was 1.7 times, only.

From the pressure dependence of *K*_b_ values, the binding volume, Δ*V*_b_, can be determined through the relation [50]:

(1) $${\left[\frac{\partial \ln K_{b}}{\partial p}\right]}_{T}=-\frac{\Delta V_{b}}{RT}.$$

**Figure 6:**
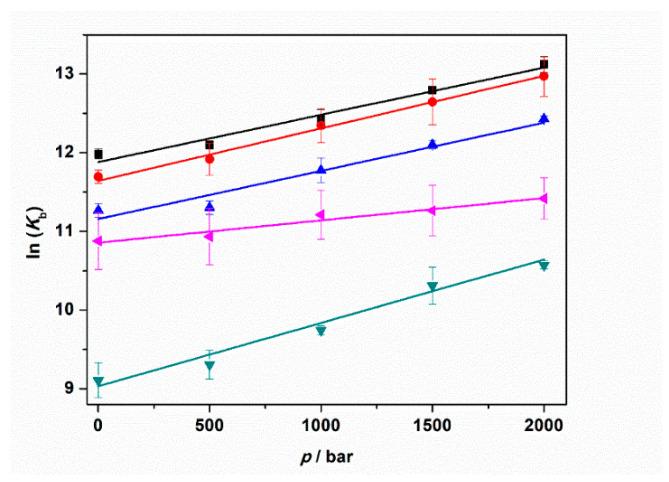
Plots of ln(*K*_b_) vs. pressure (*p*) for the complex formation between tRNA and ThT in the absence (black squares) and in the presence of 150 mM NaCl (red circles), 1 mM MgCl_2_ (blue triangles), 1 mM MgSO_4_ (reversed dark cyan triangles), and 1 mM Mg(ClO_4_)_2_ (tilted magenta triangles). All the experiments were performed in 20 mM tris-HCl buffer, 40 mM KCl, pH 6.9. The solid lines represent linear fits to the experimental data. From the slopes of ln(*K*_b_) vs. *p*, the binding volumes, Δ*V*_b_, can be determined (Equation (1)).

Here, *R* is the universal gas constant and *T* is the temperature (in K units). The binding volume, Δ*V*_b_, is defined as the difference in the partial molar volume of the tRNA–ThT complex formed, *V*_complex_, and the sum of the partial molar volumes of ThT, *V*_tRNA_, and tRNA, *V*_ThT_, i.e., Δ*V*_b_ = *V*_complex_ − (*V*_tRNA_ + *V*_ThT_). Thus, the binding volume can be directly determined by taking the slope of the plot of ln*K*_b_ vs. *p*, assuming that the volume change is independent of pressure in the (here rather small) pressure range covered. According to Le Châtelier’s principle, the pressure will favor that state which will occupy the smallest volume. Figure 6 depicts the plots of ln*K*_b_ vs. *p* for the experiments performed in the absence and in the presence of 1 mM MgCl_2_, MgSO_4_, and Mg(ClO_4_)_2_. For comparison, the corresponding plot for the experiment performed in the presence of 150 mM NaCl is also reported. All the binding volumes determined are reported in Table 3.

The binding volumes reported in Table 3 are negative for all solution conditions tested. A negative binding volume indicates that the volume occupied by the complex is smaller with respect to sum of volumes occupied by tRNA and ThT in the unbound state. In neat buffer conditions, in the presence of NaCl and MgCl_2_, the Δ*V*_b_ values are quite similar (≈−15 mL mol^−1^). Conversely, the volume change accompanying the complex formation is more pronounced (more negative) when MgSO_4_ is present in solution. Instead, a smaller volume change was determined for the perchlorate containing medium (−7.0 mL mol^−1^). Note that the structure of the tRNA molecule is pressure stable up to the maximum pressure employed in this study [25]; i.e., the reported volume changes refer exclusively to the binding process.

Finally, to fully characterize the thermodynamics of tRNA–ThT complex formation, we also determined the enthalpy change upon binding, the binding enthalpy Δ*H*°_b_, by evaluating the change in the binding constant with temperature, covering a temperature range between 5 and 25 °C. Figure S3 depicts the binding isotherms for 5, 15, and 25 °C at ambient pressure. The values of the binding constants are summarized in Table S1. According to the van’t Hoff equation, the slope of the plot of ln(*K*_b_) vs. 1/T is equal to −Δ*H*°_b_/*R*, assuming that the enthalpy change is independent of temperature (a reasonable assumption for this small temperature range):

(2) $${\left[\frac{\partial \ln K_{b}}{\partial \left(1/T\right)}\right]}_{p}=-\frac{\Delta H_{b}^{0}}{R}.$$

**Figure 7:**
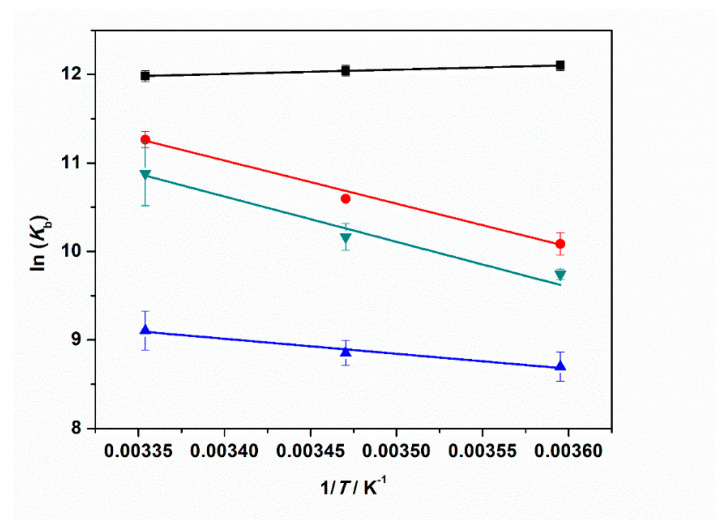
Plots of ln(*K*_b_) vs. 1/*T* for the complex formation between tRNA and ThT in the absence (black squares) and in the presence of: 1 mM MgCl_2_ (red circles), 1 mM MgSO_4_ (blue triangles), and 1 mM Mg(ClO_4_)_2_ (reversed dark cyan triangles). All the experiments were performed in 20 mM tris-HCl buffer, 40 mM KCl, pH 6.9. The solid lines represent the best fits to the experimental data. The slope of ln(*K*_b_) vs. 1/*T* is equal to -Δ*H*°_b_/*R* (Equation (2)), allowing estimation of the enthalpy change of binding, Δ*H*°_b_.

Figure 7 shows the plots of ln(*K*_b_) vs. 1/T obtained for neat buffer conditions and in the presence of 1 mM MgCl_2_, MgSO_4_, and Mg(ClO_4_)_2_. The Δ*H*°_b_ values are collected in Table 3.

Our data analysis reveals that the enthalpy change of binding is small and negative for the complex formation in neat buffer. Instead, in the presence of the Mg salts, Δ*H*°_b_ is always positive, i.e., the binding process is an endothermic event under such salt conditions. A value of ≈40 kJ mol^−1^ was found in the presence of chloride and perchlorate Mg salts. When sulfate is dissolved in the medium, the Δ*H*°_b_-value is smaller (≈14 kJ mol^−1^) but still positive. From the well-known thermodynamic relations Δ*G*°_b_ = −*RT*ln(*K*_b_) and Δ*G*°_b_ = Δ*H*°_b_ − *T*Δ*S*°_b_, we can calculate the standard binding Gibbs energy, Δ*G*°_b_, and binding entropy, Δ*S*°_b_, to obtain a full characterization of the energetics of complex formation. All thermodynamic data are reported in Table 3. The Δ*G*°_b_ values are very similar for all cases explored, pointing to the fact that the complex formation is thermodynamically favored for all salt solutions. The *T*Δ*S*°_b_ values are positive in all cases, which is most likely due to the release of hydration water surrounding the interacting partners upon binding. This is to say, enthalpy and entropy changes compensate each other to a large extent. The positive values of Δ*S*°_b_ and the positive values of Δ*H*°_b_ (or the slightly negative one in neat buffer conditions) render the formation of the tRNA–ThT complex entropy-driven.

## 3. Discussion

Various salts are known to exist in high-pressure deep subsurface environments including Earth, Mars, the icy moons of the giant gas planets, and even asteroids such as Ceres. To determine how the ions associated with these salts might influence fundamental biochemical processes and thus determine the habitability of these locations with respect to known biochemistry, we explored the effect of selected salts and high hydrostatic pressure on the complex formation between tRNA and the ligand ThT. The salts employed included MgCl_2_, MgSO_4_, and Mg(ClO_4_)_2_. We found that Mg salts strongly modulate the binding of ThT to tRNA already at rather low concentrations and in a salt-type dependent manner (Figure 1, Table 1). Already at a concentration of 1 mM Mg^2+^ salt, a significant decrease of the binding constant was observed with respect to neat buffer conditions. The decrease could be hypothesized to be due to a Debye–Hückel type of charge screening effect imposed by the presence of the salts, since ThT is positively charged and tRNA is a polyanion. However, the salt concentration is rather low, and our control experiments performed at 150 mM NaCl clearly demonstrate that this is not the case. Hence, direct binding of the magnesium salts in affecting the binding process must be invoked. On the one side, it is known that Mg^2+^ can strongly electrostatically bind to nucleic acids such as tRNA [47], in this way hampering the binding of the positively charged ThT. Differences in *K*_b_ values of the Mg^2+^ salts indicate that their anions must have an important effect as well. Hence, a key role in modulating the binding characteristics of ThT to tRNA is played by the anions, i.e., Cl^−^, SO_4_^2−^, and ClO_4_^−^, in conjunction with Mg^2+^.

Since ThT is a positively charged molecule at neutral pH, interactions of the anions with the ligand, thereby decreasing the activity of the ligand, can be expected to contribute to the observed decrease of *K*_b_ as well. From the data reported in Table 1, the binding affinity of ThT for tRNA in the presence of Mg^2+^ salts follow the order: Cl^−^ > ClO_4_^−^ > SO_4_^2−^, indicating an increasing affinity of the anion to the ligand in this order. The sulfate is a divalent anion, which is characterized by a high negative surface charge density [51]. Consequentially, pronounced binding of the anion to ThT can be expected. The perchlorate anion has a smaller charge density and a slightly hydrophobic character, thereby taking an intermediate place in this order of anions.

At neat buffer conditions, a slightly negative Δ*H*°_b_ value was determined, which was coupled with a positive binding entropy, Δ*S*°_b_ (Table 3). The negative Δ*H*°_b_ value is probably due to the non-covalent interactions established between ThT and tRNA, invoking both electrostatic (with phosphate groups) and hydrophobic (stacking with nucleobases) interactions. The positive Δ*S*°_b_ can be ascribed to the release of hydration water surrounding the interacting partners [52]. Conversely, in the presence of Mg^2+^ salts, Δ*H*°_b_ is always positive, indicating that some weak bonds are broken during complex formation. Such an effect could be due to a combination of several factors: the dissociation of anions previously bound to ThT, the release of tRNA-bound Mg^2+^ occupying the binding site, and hydration changes accompanying these processes prior to ThT binding to tRNA, which is connected with (partial) desolvation of the interacting partners.

Applying HHP fluorescence spectroscopy, it was possible to perform a volumetric analysis of complex formation (Table 3). We found that for all solution conditions studied, the binding volume, Δ*V*_b_, is negative, revealing that the complex occupies a smaller volume with respect to the uncomplexed state. This is to say, the application of pressure favors the formation of the complex. We note that the volume changes observed here are very small, in the order of one H_2_O molecule, only (*V*_H2O_ = 18 mL mol^−1^). Depending on the salt type, pressurization up to 2 kbar increased *K*_b_ by a factor of 2–4 (Table 2). The negative binding volumes could be ascribed to a small decrease of void volume upon ThT binding to tRNA, which might be due to a reduction of volume fluctuations of the macromolecule upon binding, which lead to a tightening of internal atomic packing of the tRNA. Another small contribution to the decrease of Δ*V*_b_ could come from the ligand ThT itself, as there is a decrease in its conformational degrees of freedom upon binding (by blocking the rotation of the two aromatic planes of ThT, which are connected by a single bond).

Complex formation between two interacting partners is generally accompanied by (partial) dehydration [52]. This is consistent with a corresponding positive value of Δ*S*°_b_ which has in fact been observed. Generally, water bound to polar and charged groups (such as to phosphates in nucleic acids) occupies a smaller volume with respect to bulk water due to electrostrictive effects (i.e., the contraction of the hydration shell around the biomolecules with densities higher than those of bulk water) [53]. For example, the unfolding of G-quadruplexes is accompanied by water uptake, which contributes negatively to the overall change in the unfolding volume [54]. However, consequently, the dehydration process upon binding of the ligand to tRNA should be characterized by a small positive volume change that, in our case, cannot compensate the packing factors contributing to Δ*V*°_b_. Variations of Δ*V*_b_ of the different Mg^2+^ salts may be due to modulation of the hydration contribution in the presence of the different anions, which change the water structure by electrostriction (most pronounced for SO_4_^2−^ and least or even reversed for the chaotrope ClO_4_^−^ [51]) to a different extent upon dissociation from the ligand upon binding. This effect may also cause the less pronounced increase of the binding constant upon pressurization when perchlorate is released into the bulk solution.

With respect to the astrobiological consequences, the local concentrations of ions in any given location will depend upon the local deposition of ions and the degree of dilution. The concentration of perchlorates varies a fair bit across the surface of Mars. For example, Hecht et al. [29], investigating perchlorate concentrations in the Martian soils in the northern latitudes, found that leachates contained 10 mM of dissolved salts with 0.4 to 0.6% perchlorate by mass leached from samples. This concentration is higher than the 1 mM at which we observed reduced ligand binding. The concentration of perchlorate would be lower where ions were dissolved in larger bodies of water than the confined experimental chamber of the lander’s wet chemistry lab. Nevertheless, the data from Mars show that the effects we observe are broadly consistent with potential environmentally relevant concentrations.

## 4. Materials and Methods

### 4.1. Materials

The nucleic acid tRNA from *E. coli* (mean molecular weight of 25 kDa) was purchased from Sigma Aldrich Chemicals (Taufkirchen, Germany) and used without further purification. The fluorescence molecule Thioflavin T (ThT: 4-(3,6-dimethyl-1,3-benzothiazol-3-ium-2-yl)-*N,N*-dimethylaniline chloride), the salts sodium chloride (NaCl), potassium chloride (KCl), magnesium chloride (MgCl_2_), magnesium sulfate (MgSO_4_), magnesium perchlorate (Mg(ClO_4_)_2_), and tris(hydroxymethyl)aminomethane hydrochloride (Tris-HCl) for buffer preparation were also obtained from Sigma Aldrich Chemicals. Deionized water was used for all buffer and sample preparations.

### 4.2. Sample Preparation

Concentrated stock solutions of the fluorescent probe (ThT) and the nucleic acid (tRNA) were prepared by dissolving the lyophilized powder in the pressure stable buffer (20 mM Tris-HCl, 40 mM KCl) with a final pH of 6.9. The exact concentrations were spectrophotometrically determined by means of a UV-1800 spectrometer from Shimadzu Corporation (Kyoto, Japan). For ThT, the concentration was determined by evaluating the absorbance at 412 nm, using the extinction coefficient *ε*(412 nm) = 36,000 M^−1^ cm^−1^ [55]. For the tRNA, the concentration was determined by evaluating its absorbance at 260 nm and considering that 1 mg of dry substance dissolved in 1 mL corresponds to 16 *A*_260_ units, as reported by the manufacturer. Buffers containing salts (NaCl, KCl, MgCl_2_, MgSO_4_, and Mg(ClO_4_)_2_) were prepared by dissolving an appropriate amount of salt in the Tris-HCl buffer.

### 4.3. Steady-State Fluorescence Spectroscopy

Steady-state fluorescence spectroscopy was used to follow the interaction between Thioflavin T and tRNA. A series of solutions with fixed concentration of 0.5 µM ThT were prepared, and the concentration of tRNA was varied between 0 and ≈70  µM, depending on the medium conditions. The samples were mixed for 15 min at 900 rpm before the measurements. Fluorescence emission spectra were recorded by means of a K2 fluorometer from ISS, Inc. (Champaign, IL, USA). The excitation wavelength was set to 438 nm. The emission was collected from 460 to 560 nm. The width slits for both the excitation and emission monochromators were set to 8 nm. Measurements at ambient pressure were performed by using a 0.3 cm path length quartz cuvette at temperatures of 5, 15, and 25 °C. The temperature was controlled by a circulating water bath directly connected to the sample holder. For the pressure-dependent experiments, an ISS high-pressure cell system and cylindrical quartz cuvettes were used. A pressure range from 1 to 2000 bar was covered. The pressure was controlled by means of a manual pump and water was used as pressurizing fluid. The samples were loaded in the cuvette, sealed with DuraSeal™ laboratory stretch film, and placed into the high-pressure cell. High-pressure experiments were performed at the temperature of 25 °C. The temperature was controlled by means of a water circulating bath directly connected to the high-pressure cell. For the determination of the binding constants (*K*_b_), a plot of Δ*F* vs. total concentration of tRNA was made. The experimental data were fitted according to a 1:1 binding model as previously described [41]:

(3) $$\Delta F=\left[\frac{1}{\left[S_{0}\right]}\left(-\frac{\;\sqrt{b^{2}-4ac}-b}{2a}\right)\right]{\left(\Delta F\right)}_{\mathrm{total}}.$$

Δ*F* is the difference between *F* and *F*_0_, where *F* is the ThT fluorescence intensity in the presence of tRNA and *F*_0_ is the intensity in the absence of tRNA, ${\left(\Delta F\right)}_{\mathrm{total}}$ is the maximum difference between *F* and *F*_0_, and [*S*_0_] is the total concentration of ThT (kept constant during the titration). In this equation, *a* = *nK*_b_, *b* = (1 *+ n*[S_0_]*K*_b_ + [L_0_]*K*_b_), and *c* = [S_0_][L_0_]*K*_b_, where *n* is the stoichiometry set to the value of 1, [L_0_] is the total tRNA concentration (which is varied during the titration), and *K*_b_ is the binding constant. The reported binding constants represent the average of three independent experiments.

### 4.4. Circular Dichroism Spectroscopy

To study the secondary structure adopted by the tRNA in the different media and in the presence of ThT (1 mM), circular dichroism (CD) spectroscopy was employed. The CD spectra were recorded by means of a Jasco J-715 spectropolarimeter (Jasco Corporation, Tokyo, Japan). CD spectra of 20 µM tRNA solutions were recorded in the range of 300–190 nm using a 0.1 cm thick quartz cuvette. The instrument parameters were set as follows: scan rate of 50 nm min^−1^, response time of 2 s, and bandwidth of 4 nm. A background blank (pure buffer or saline buffer in the absence or presence of ThT) was subtracted from each sample.

## 5. Conclusions

We have shown that the Mg^2+^ salts MgCl_2_, MgSO_4_, and Mg(ClO_4_)_2_, which are widely distributed on Mars, and the MgCl_2_ and MgSO_4_ salts, which are present in the interiors of icy moons, some asteroids, and the deep subsurface of Earth, significantly reduce the binding propensity of ThT to tRNA. However, at mM concentrations, binding is still favored. This detrimental effect is largely due to the interaction of ThT with the salt anions, which leads to a sharp decrease in the activity of the ligand (i.e., a decrease in its activity coefficient). The thermodynamic driving force changes in the presence of the Mg^2+^ salts and becomes more entropically driven in the presence of the Mars salts. Remarkably, an increase in hydrostatic pressure favors ligand binding regardless of the salt type. Thus, although the binding constant is reduced, the harsh salt and pressure conditions in the deep subsurface of planetary bodies do not necessarily preclude the nucleic acid–ligand interactions studied here. The binding of small ligands and proteins to other RNA types (such as rRNA, mRNA, and non-canonical nucleic acid structures) should be explored as well in order to assess the role of sequence and conformation on the complex formation in the presence of Mars relevant salts. Such studies will be part of future projects.

## Figures and Tables

**Table 1:** Binding constants, *K*_b_, for tRNA–ThT complex formation in 20 mM Tris-HCl buffer, 40 mM KCl, pH 6.9, in the absence and in the presence of the indicated salts at the temperature of 25 °C.

Solution Conditions	*K*_b_/10^6^ M^−1^
Tris-HCl 20 mM, 40 mM KCl, pH 6.9	0.16 ± 0.01
+150 mM NaCl	0.12 ± 0.01
+1 mM MgCl_2_	0.078 ± 0.010
+1 mM MgSO_4_	0.009 ± 0.002
+1 mM Mg(ClO_4_)_2_	0.053 ± 0.019

**Table 2:** Binding constants, *K*_b_, for tRNA–ThT complex formation in 20 mM Tris-HCl buffer, 40 mM KCl, pH 6.9, in the absence and in the presence of the indicated salts at the temperature of 25 °C and at the indicated pressures.

Solution Conditions	*p*/bar	*K*_b_/10^6^ M^−1^
Tris-HCl 20 mM, 40 mM KCl, pH 6.9	1	0.16 ± 0.01
	500	0.18 ± 0.01
	1000	0.25 ± 0.03
	1500	0.36 ± 0.01
	2000	0.50 ± 0.05
+150 mM NaCl	1	0.12 ± 0.01
	500	0.15 ± 0.03
	1000	0.23 ± 0.05
	1500	0.31 ± 0.09
	2000	0.43 ± 0.11
+1 mM MgCl_2_	1	0.078 ± 0.007
	500	0.081 ± 0.007
	1000	0.13 ± 0.02
	1500	0.18 ± 0.01
	2000	0.25 ± 0.01
+1 mM MgSO_4_	1	0.009 ± 0.002
	500	0.011 ± 0.002
	1000	0.017 ± 0.001
	1500	0.030 ± 0.007
	2000	0.039 ± 0.002
+1 mM Mg(ClO_4_)_2_	1	0.053 ± 0.019
	500	0.056 ± 0.020
	1000	0.074 ± 0.023
	1500	0.078 ± 0.025
	2000	0.091 ± 0.024

**Table 3:** Binding constants, *K*_b_, for tRNA–ThT complex formation in 20 mM Tris-HCl buffer, 40 mM KCl, pH 6.9, in the absence and in the presence of the indicated salts at the temperature of 25 °C.

Solution Conditions	*K*_b_*/*10^6^ M^−1^	Δ*G*°_b_/kJ mol^−1^	Δ*H*°_b_/kJ mol^−1^	*T*Δ*S*°_b_/kJ mol^−1^	Δ*V*_b_/mL mol^−1^
Tris-HCl 20 mM, 40 mM KCl, pH 6.9	0.16 ± 0.01	−29.7 ± 0.2	−4.1 ± 0.2	25.6 ± 0.2	−14.9 ± 1.3
+150 mM NaCl	0.12 ± 0.01	−29.0 ± 0.2	N.D. *	N.D. *	−16.6 ± 0.8
+1 mM MgCl_2_	0.078 ± 0.007	−27.9 ± 0.2	40.5 ± 2.2	68.4 ± 2.2	−15.2 ± 2.1
+1 mM MgSO_4_	0.009 ± 0.002	−22.6 ± 0.6	14.2 ± 2.0	36.7 ± 2.1	−19.9 ± 1.9
+1 mM Mg(ClO_4_)_2_	0.053 ± 0.019	−27.0 ± 0.9	42.6 ± 6.7	69.6 ± 6.7	−7.0 ± 0.9

* Not determined.

## Data Availability

The data presented in this study are available on request from the corresponding authors.

## Supplementary Materials

The following are available online at www.mdpi.com/article/10.3390/ijms221910861/s1.
