# Supplementary material for: Ions in the Deep Subsurface of Earth, Mars, and Icy Moons: Their Effects in Combination with Temperature and Pressure on tRNA–Ligand Binding
Source: Int J Mol Sci. 2021 Oct 8;22(19):10861. doi: 10.3390/ijms221910861 (PMC8509373; doi:10.3390/ijms221910861)
Supplement: Supplementary file 1 [file ijms-22-10861-s001.zip › ijms-1401376-supplementary.pdf]

Supplementary Information

Ions and high pressure in the deep subsurface of Earth, Mars and icy moons: their effects in combination with temperature and pressure on tRNA-ligand binding

Nisrine Jahmidi-Azizi [1], Stewart Gault [2], Charles S. Cockell [2], Rosario Oliva [1,*], and Roland Winter [1,*]

[1] Physical Chemistry I - Biophysical Chemistry, Department of Chemistry and Chemical Biology, TU Dortmund University, 44227 Dortmund, Germany; nisrine.jahmidi@tu-dortmund.de

[2] UK Centre for Astrobiology, SUPA School of Physics and Astronomy, University of Edinburgh, James Clerk Maxwell Building, Edinburgh EH9 3FD, UK; s.a.gault@sms.ed.ac.uk (S.G.); c.s.cockell@ed.ac.uk (C.S.C.)

* Correspondence: rosario.oliva@tu-dortmund.de (R.O.); roland.winter@tu-dortmund.de (R.W.)

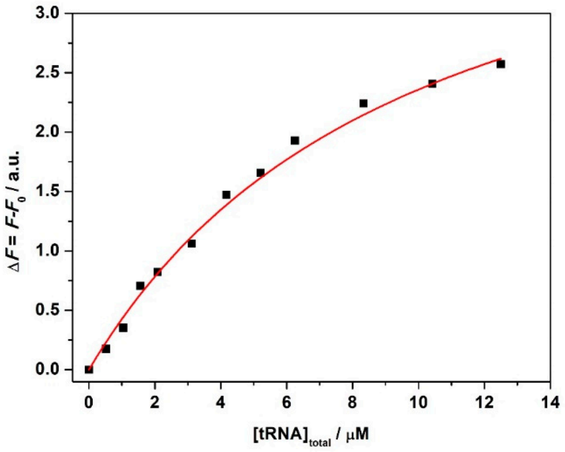

Figure S1. Binding isotherm for complex formation between tRNA and ThT obtained at the temperature of 25 °C and pressure of 1 bar in the presence of 150 mM NaCl. The solid line represents the best fit to th experimental data according to 1:1 binding model. The binding isotherms were obtained by plotting $\Delta F = F\text{-}F_0$ *vs*. tRNA total concentration. Here, F and F_0 are the ThT fluorescence intensities in the presence and in the absence of tRNA, respectively. The experiment was performed in 20 mM Tris-HCl buffer, 40 mM KCl, pH 6.9.

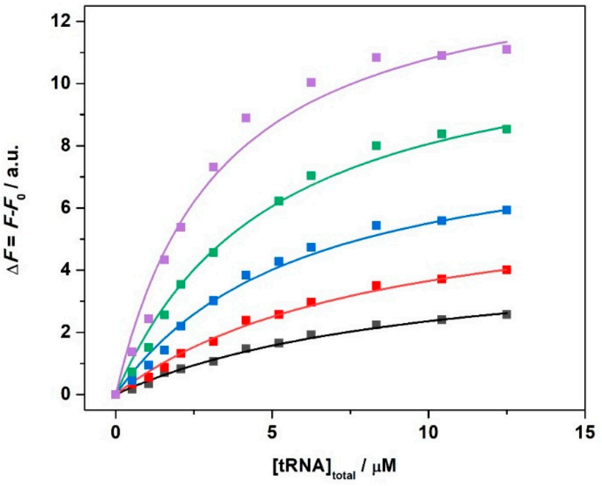

Figure S2. Binding isotherms for complex formation between tRNA and ThT obtained at the temperature of 25 °C in the in the presence of 150 mM of NaCl, at pressures of: 1 bar (black squares), 500 bar (red squares), 1000 bar (blue squares), 1500 bar (green squares), and 2000 bar (magenta squares). The solid lines represent the best fit to experimental data according to a 1:1 binding model. The binding isotherms were obtained by plotting $\Delta F = F\text{-}F_0$ *vs.* tRNA total concentration. Here, F and F_0 are the ThT fluorescence intensities in the presence and in the absence of tRNA, respectively. All the experiments were performed in 20 mM Tris-HCl buffer, 40 mM KCl, pH 6.9.

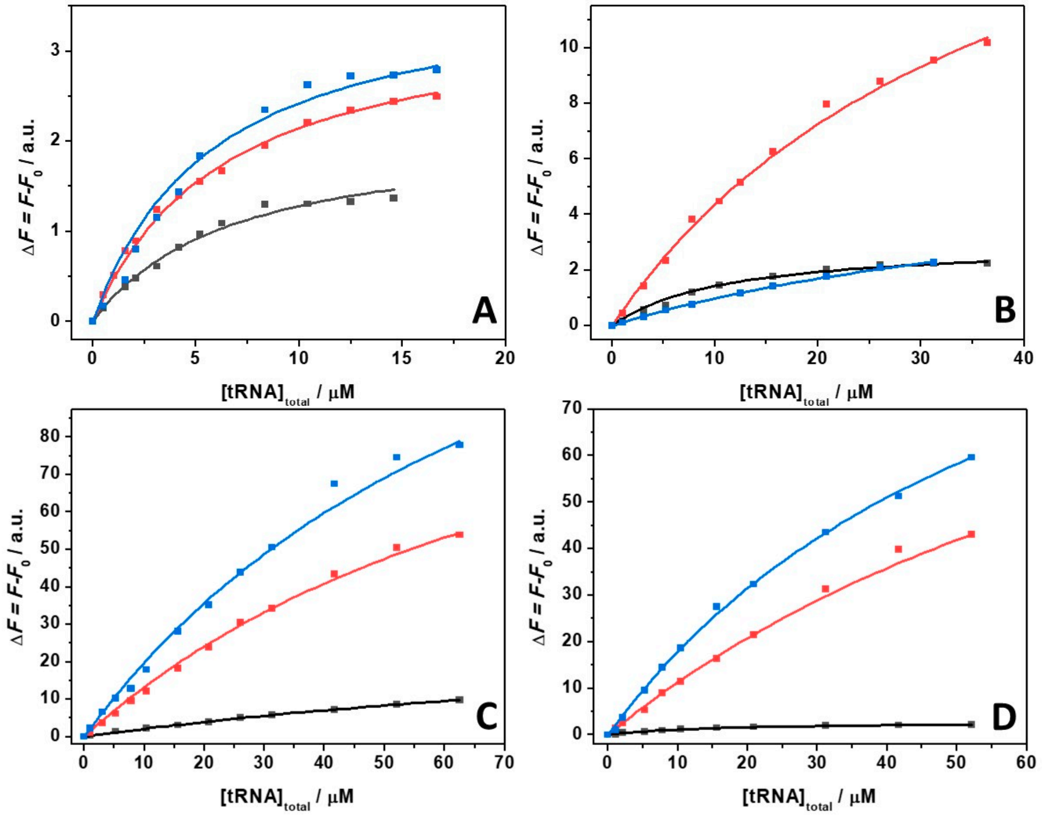

Figure S3. Binding isotherms for complex formation between tRNA and ThT obtained at the pressure of 1 bar and at the temperatures of 25 °C (black squares), 15 °C (red squares) and 5 °C (blue squares) in the absence (A) and in the presence of 1 mM of: $MgCl_2$ (B), $MgSO_4$ (C) and $Mg(ClO_4)_2$ (D). The solid lines represent the best fit to the experimental data according to a 1:1 binding model. The binding isotherms were obtained by plotting $\Delta F = F\text{-}F_0$ *vs.* tRNA total concentration. Here, F and F_0 are the ThT fluorescence intensities in the presence and in the absence of tRNA, respectively. All the experiments were performed in 20 mM Tris-HCl buffer, 40 mM KCl, pH 6.9.

Table S1. Binding constants (K_b) for the tRNA/ThT complex formation in 20 mM Tris-HCl buffer, 40 mM KCl, pH 6.9, in the absence and in the presence of the indicated salts at temperatures of 5 °C, 15 °C and 25 °C and p = 1 bar.

Solution Conditions	T / °C	K_b / 10^6 M^{-1}
Tris-HCl 20 mM, pH 6.9	5	0.18 ± 0.1
Tris-HCl 20 mM, pH 6.9	15	0.17 ± 0.1
Tris-HCl 20 mM, pH 6.9	25	0.16 ± 0.1
+ 1 mM $MgCl_2$	5	0.024 ± 0.003
+ 1 mM $MgCl_2$	15	0.040 ± 0.001
+ 1 mM $MgCl_2$	25	0.078 ± 0.007
+ 1 mM $MgSO_4$	5	0.006 ± 0.001
+ 1 mM $MgSO_4$	15	0.007 ± 0.001
+ 1 mM $MgSO_4$	25	0.009 ± 0.002
+ 1 mM $Mg(ClO_4)_2$	5	0.017 ± 0.001
+ 1 mM $Mg(ClO_4)_2$	15	0.026 ± 0.004
+ 1 mM $Mg(ClO_4)_2$	25	0.053 ± 0.019
